# Remembering What We Always Knew: Development of an Indigenous Diabetes Prevention Model

**DOI:** 10.1016/j.cdnut.2025.104570

**Published:** 2025-02-19

**Authors:** Baiwen Peng, Kate Welshons, Trina Adler, Hyunjun Kim, Elder June M Blue

**Affiliations:** Department of Family, Health and Wellbeing, University of Minnesota Extension, St. Paul, MN, United States

**Keywords:** Indigenous, traditional ecological knowledge, culturally relevant, two-eyed seeing, diabetes prevention, cultural approaches, chronic disease prevention

## Abstract

**Background:**

Although the National Diabetes Prevention Program in the United States demonstrates success in physical health improvements, it lacks efficacy for recruiting and retaining Indigenous participants. In response to its shortcomings, diabetes prevention initiatives that integrate Indigenous traditional ecological knowledge emerged. Nevertheless, these initiatives also face various challenges that undermine their connections to authentic Indigenous needs, and their developments within the context of mainstream organizations are laden with complexity.

**Objectives:**

The purpose of this initiative was to develop a culturally relevant diabetes prevention program for Indigenous women in the United States aged 40 and older that would address challenges to existing programs.

**Methods:**

Informed by a detailed analysis of culturally relevant diabetes prevention programs in the United States and the Two-Eyed Seeing approach, the University of Minnesota Extension gathered an advisory council of Indigenous women-Elders to cocreate and field-test a culturally centered program model. Extension supported the process through sharing literature review findings, creating connections to national expertise in Indigenous diabetes prevention, and facilitating participatory evaluation.

**Results:**

The initiative facilitated reconnection with Indigenous cultural practices and healthy living traditions through Indigenous activities. The program provided a sisterhood of support for the women as they explored and reclaimed their cultural identities. Outcomes reported by participants included increased sense of belonging, increased reliance on cultural ways for healthy living, increased capability to engage in cultural practices, increased confidence in using cultural medicines for health and healing for self and others, and improved confidence in Elder status.

**Conclusions:**

Indigenous cultural approaches to health promotion emphasize community values, offer a holistic approach to health that integrates mental, physical, emotional, and spiritual well-being, and consciously recognize historical trauma and racism. Applying the Two-Eyed Seeing approach to program development can successfully integrate Indigenous cultural approaches to health promotion in the context of mainstream systems.

## Introduction

By prohibiting traditional ecological knowledge and practices, systemic oppression has played a direct role in how Indigenous communities have been disproportionately affected by diabetes and other chronic conditions in the United States [1]. Mainstream health and nutrition programs, such as the National Diabetes Prevention Program, often center on individualism, primarily address physical health, often employ an expert model, rely on Western scientific principles, and lack a historical perspective, typically operating under the assumption of a nonracist environment [[2], [3], [4]].

These programs do not align with Indigenous culture, which focuses on multiple dimensions of health. In an Indigenous worldview, physical health, which is primarily emphasized in Western models, is one dimension alongside spirituality, intergenerationality, and a manifestation of fate [5,6], among others. These additional dimensions are rarely referenced in the dominant framework.

Such misalignment could potentially explain why mainstream programs suffer low enrollment and retention among Indigenous communities [7]. These programs do not provide a familiar or comfortable environment where Indigenous communities can effectively reflect on their health and address health problems in a way that resonates with their culture and tradition [8].

In addition, mainstream programs do not emphasize how Indigenous individuals experience the programs. Although these programs produce significant positive outcomes in terms of improved health indicators [9], they fail to consider and report if collecting health metrics such as blood pressure and BMI cause discomfort or reinforce historical trauma.

Over the past 3 decades, diabetes prevention programs tailored to Indigenous communities in the United States have emerged. Their emergence may have been inspired by the rise of community-based participatory research [10] and a decolonization agenda in public health [11]. These developments in public health and social sciences focus on the empowerment of Indigenous communities and feature a reconsideration of their culture in response to Western sciences.

Research on these culturally relevant diabetes prevention programs has shown significant improvements across various health indicators, attesting to the significant role of culture in diabetes prevention for Indigenous individuals [12]. A careful review of these programs shows that they are not without shortcomings with which our program team grappled in determining ways to move forward with the initiative discussed in this article.

The next section explores the historical development of culturally relevant diabetes prevention programs for Indigenous communities in the United States and reveals their improvements made over time as well as shortcomings. Based on this analysis, the Remembering What We Always Knew (RWWAK) project was designed using Two-Eyed Seeing, an approach that integrates Indigenous and Western ways of knowing [13,14] and resonates with the throughline of existing programs. Details of RWWAK and Two-Eyed Seeing are included in the third section. The fourth section examines the result of an evaluation of RWWAK, followed by conclusions and discussions.

### The stages of existing culturally relevant diabetes prevention programs for Indigenous communities in the United States

A detailed analysis of culturally relevant diabetes prevention programs for Indigenous communities in the United States indicates that these programs vary on different fronts and the degree to which they resonate with Indigenous culture differs. Despite their diversity, there are throughlines that categorize the programs into several stages, each representing distinct program characteristics. The stages also indicate a transition from less to more cultural relevance manifested in the programs ranging from the 1990s to the present day. This pattern is not necessarily linear, and programs at different stages of cultural relevance coexist today. The coexistence of the diverse forms suggests that practitioners and academics working on these programs may not be well informed of each other’s work and, instead, developed their projects independently, suggesting an urgent need to map out the trajectories of these programs as a repository that informs the best practices.

The following paragraphs illustrate 3 stages of diabetes prevention programs for Indigenous communities in the United States. They are distinguishable along the lines of community participation and compatibility with Indigenous cultures. Table 1 summarizes key dimensions of the stages with examples and shows a developmental pattern [[15], [16], [17], [18], [19], [20], [21], [22], [23], [24]].

**TABLE 1:** Stages of culturally relevant diabetes prevention programs for indigenous communities in the united stages.

Project name	Community advisory board	Program design	Program implementation	Evaluation	Participant recruitment
Stage 1: "A goodwill expert":top-down interventions					
The Diabetes Prevention Program of the Alaska Siberia Project [15]	X	X			
Stage 2: A persisting intervention model					
Special Diabetes Program for Indians [16]	X	X	X		
PILI ‘Ohana Project [17]		X	X		
Life in BALANCE [18]		X	X		
Stage 3: The emergence of organic Indigenous programming					
Enhanced Diabetes Prevention Program [19]	X	X	X		
Together Overcoming Diabetes [20]	X	X	X		
Balance Your Life with Diabetes [21]	X	X	X		
Group Gardening [22]	X	X	X		
Tribal Food Project [23]	X	X	X	X	X
Native Diabetes Wellness Program [24]	X	X	X		

Project name	Indigenous food/cooking	Physical activity	Indigenous ceremony	Indigenous storytelling	Gift sharing

Stage 1: "A goodwill expert":top-down interventions					
The Diabetes Prevention Program of the Alaska Siberia Project [15]	X				
Stage 2: A persisting intervention model					
Special Diabetes Program for Indians [16]	X	X			
PILI ‘Ohana Project [17]	X				
Life in BALANCE [18]	X	X		X	
Stage 3: The emergence of organic Indigenous programming					
Enhanced Diabetes Prevention Program [19]			X	X	
Together Overcoming Diabetes [20]	X	X		X	X
Balance Your Life with Diabetes [21]	X		X	X	
Group Gardening [22]	X	X	X		
Tribal Food Project [23]		X	X	X	X
Native Diabetes Wellness Program [24]	X			X	

Columns in the first part of the table describe the ways in which Indigenous communities are involved in the programs. An “X” for “Program Design,” for example, means that Indigenous communities are involved in the design of the program. The second part of the table shows cultural elements included in the programs.

Abbreviation: PILI, Partnerships to Improve Lifestyle lnterventions.

#### Stage 1: “A goodwill expert”: top-down interventions

Diabetes prevention designed specifically for Indigenous communities emerged in the 1990s. At this stage, the programs represented the “goodwill” of health experts as they recognized that Indigenous communities might have special health-related needs, which distinguished them from other minority ethnic, cultural groups. However, understanding of such needs lacked depth, and the health experts were essentially “foreign” to authentic Indigenous culture, and their programs were culturally irrelevant to tribal communities, leaving urgent community needs unmet.

Examples include the Diabetes Prevention Program of the Alaska Siberia Project [15]. Although efforts were made within this initiative to address cultural differences, Indigenous community members did not provide leadership for the programs, and activities that health experts started were modeled after Western scientific health prototypes such as workshops and counseling, despite attention paid to the promotion of Indigenous cultural practices [15,25]. Additionally, evaluations of these programs were based on Western notions of health and standards, focusing on indicators such as blood pressure and BMI.

Programs as such carried a slight connotation of imposition, and the goodwill experts, without sufficient knowledge of Indigenous culture, introduced to Indigenous communities a set of Western scientific terminologies, principles, and testing setup, which did not resonate with the Indigenous vocabulary. Although these programs delivered positive health behavior and biometric results [15,25], what the Indigenous communities thought about their experiences and their understanding of the results remained unaddressed. This is a critical issue as ensuring a culturally resonant participant experience has great potential for positive impacts on recruitment, retention, and sustainable health results.

#### Stage 2: a persisting intervention model

A major shift evident in Stage 2 programs is the use of community-based participatory approaches. The rise of this approach is considered progressive as it started to consider the diverse and heterogeneous needs within Indigenous communities. This trend also signals a new attitude of Western experts regarding Indigenous communities: they acknowledge their outsider roles, not proficient in the local culture or language, and that health expertise lies within the community. As a result, Indigenous community members are increasingly involved in diabetes prevention.

Compared with Stage 1, Stage 2 programs are mildly (but still) interventional. They utilize the vocabulary of “interventions” and feature curriculum-based activities. Indigenous Elders might be involved as consultants to make sure that the course content is culturally appropriate, but the overall form the programs take, as organized education sessions, may be considered out of place in Indigenous culture. Although Indigenous elements are incorporated into the programs, which signals progress, the programs remain deeply rooted in Western scientific conventions that highlight experimental design, curriculum as intervention, and health measured by scientific indicators.

An example from this category is the Life in BALANCE project [18]. Although the project is participatory, involving Indigenous communities as collaborators, its activities are built on educational interventions; additionally, its evaluations focus specifically on measures that are narrow in scope and align with Western conceptualizations of health.

Although these programs have delivered positive behavioral and biometric outcomes [17], they have not sufficiently considered how the Indigenous communities have experienced the programs and have overlooked potential discomfort and cultural mismatch.

#### Stage 3: the emergence of organic Indigenous programming

Compared with Stage 2, programs at this stage organically connect with the everyday life of Indigenous communities and align with Indigenous traditions. For example, the program reported by Rosas et al. [19] features activities such as storytelling, an essential culturally-based activity through which individuals learn from and support each other. Similar elements are found in the project by Brown et al. [22] that invites participants to garden, providing them with an opportunity to be reconnected with nature and the land consistent with Indigenous culture. Another example is the Traditional Foods Project [23] that incorporates significant Indigenous elements, such as gardening, storytelling, and traditional food. As with Stage 2, programs at Stage 3 commonly adopt a Western evaluative approach. These programs have produced positive health outcomes, demonstrating that cultural relevance contributes to diabetes prevention [19,22].

The progression through the stages indicates a turn toward an organic, rather than interventional, participatory approach that centers Indigenous community voices and needs and diminishes the use of expert-model influences typically found in mainstream health promotion programs. Nevertheless, Stage 3 programs still suffer from 3 weaknesses:

1) The persisting Western understanding of health. Health is primarily understood as physical, measured by Western indicators, whereas other dimensions of health (for example, spiritual) in the Indigenous culture are largely overlooked.
2) Western approach to evaluations. In most cases, it is not up to the Indigenous community to decide whether the programs are effective or not. The outcomes could meet Western expectations but disrupt Indigenous notions of health. As normative practices, evaluations as such potentially lead to recolonization.
3) Overlooking Indigenous division of labor. These programs fail to respect health-related roles traditionally played by Indigenous individuals. In many Indigenous cultures, Elder women are considered medicine keepers and key catalysts for health promotion. To align with such tradition, diabetes prevention should support Elder women in developing and sharing such skills and playing traditional roles. Program implementation that respects and engages traditional roles and division of labor is rare, and a one-size-fits-all approach is common, which reflects Western notions of individual responsibility.

Recognizing the strengths and weaknesses of existing culturally relevant diabetes programs for Indigenous communities, the program team developed RWWAK, which is tailored to Indigenous communities, particularly Indigenous women, in mid-western United States. Development relied upon community wisdom and leadership, resulting in a program that emphasizes community values and holistic views of health that integrate mental, physical, emotional, and spiritual well-being, consciously recognizes historical trauma and racism, honors community wisdom and traditional ecological approaches, and utilizes the rich resources of Indigenous knowledge about healthy living [22,26]. Evaluation of the program, accordingly, generated results that aligned with the Indigenous notion of health. Given these characteristics, RWWAK extends the existing models exhibited by Stage 3 programs.

## Methods

### RWWAK’s rationale and approach

With funding from the Centers for Disease Control and Prevention via the Minnesota Department of Health, the University of Minnesota Extension (Extension) was contracted to facilitate the development of a culturally-based diabetes prevention program for Indigenous women. The intentional systemic and practical shifts that occurred resulted in a balance that respected and revitalized Indigenous traditions, and the program was designed to be an alternative to the National Diabetes Prevention Program, which has less efficacy with regard to recruitment and retention for Indigenous communities in the United States [27]. With heavy emphasis on Indigenous communities and culture, Extension turned over funding and resources to 2 respected Indigenous women-Elders, one of which is an Extension staff member, and the other a contracted Indigenous Elder. The 2 Elders established an advisory committee of Indigenous women to develop and field-test a program model. This team of Indigenous women also served as an ethics committee regarding the ways the project was envisioned, implemented, and evaluated to ensure culturally respectful and resonant practice.

The advisory committee and Elders identified salient content and a program framework through culturally relevant events and meetings in which they shared stories about their lives, families, cultures, and values, all stemming from lived experience. Because not all were from the same nation, over the course of the program, different heritages and traditions were respected. Leveraging these foundational cultural elements, a distinctive and culturally attuned program model was created that supports women in embracing their cultural identities as medicine keepers, and claiming holistic health through reconnection with Indigenous cultural practices and healthy living traditions.

This work was based on Two-Eyed Seeing, an approach that utilizes the best of both Western and Indigenous knowledges and cultures to deepen understanding and solve problems [13,14]. Over the last 2 decades, it has been extensively used in educational and academic settings [14]. Although firmly rooted in community, an academic approach was integrated, including identifying and sharing relevant journal articles, consulting with expertise in Indigenous health promotion and diabetes prevention work, and determining meaningful benchmarks measured through culturally appropriate evaluation processes.

RWWAK at the University of Minnesota was exempt from the University of Minnesota Institutional Review Board’s review because it is considered an evaluation rather than research.

### Theory of change

The Elders and advisory team developed a theory of change that eschewed traditional aspects of mainstream disease prevention to instead fully embrace the role culture plays in promoting health and well-being. Specifically, diabetes was not mentioned within the program, and activities were not focused on health skills and behaviors aimed at reducing BMI, utilizing mainstream nutrition science and increasing physical activity habits to prevent diabetes. For the Indigenous Elders and advisory team, focusing on specific diseases was not as relevant as prevention of disease and protection of health through honoring and respecting the gifts of the Creator and “true ways.” In their cultures, health was defined holistically, and illness was considered a manifestation of lack of respect for/practice of cultural ways, largely influenced by dominant culture.

For many Indigenous communities, Elder Indigenous women play important roles and responsibilities in their families and communities as medicine keepers. They provide essential medicines and wisdom to fulfill the responsibility of maintaining the health of the community; as in all things they are responsible to their ancestors, their community, and their children. With this in mind, the women developed the following theory of change for their program:

Through participation in transformative culturally rooted activities and opportunities for sharing and reflecting on joys, vulnerabilities, and experiences in the context of a supportive society of Indigenous women, members will reclaim and reconnect to their cultural roles as Elders and medicine women. This sense of cultural identity and purpose will lead members to understand and internalize their own value, to care for their own health and well-being, and to mentor others to do so.

### Program characteristics and activities

Through the course of just over 1 year, advisory committee members planned and participated in a series of culturally-based experiences and ceremonies, as described in Table 2, and shared their learnings with their families and communities.

**TABLE 2:** Program activities and learnings/cultural values.

Activity	Learnings/cultural values
Recruitment	• The Elders offered sacred tobacco to their ancestors for guidance on who to invite to the program, out of respect and gratitude
Presession. Welcome breakfast	• Introduced members to the program; focused on responsibilities and commitment involved • Opportunity to get comfortable and build trust together in shared space • Acknowledged that not all are from the same nation, (although often Indigenous people get “grouped” together) and different heritages and traditions are respected • Women were given traditional gifts
Session 1. Medicine making	• Learned to make cultural medicines and enjoyed cultural foods • Women shared wisdom from their own nations • Focused on healing and honoring the space the women shared
Session 2. Wild ricing	• Overnight stay in a yurt was a bonding experience for the women • Connected women to a global perspective on the sacredness of water • Emphasized the importance of treating the earth with intention and care
Session 3. Cooking traditional foods, art therapy, and overnight stay	• Cooked and shared traditional foods • Participated in art therapy with a focus on trees • Discussed how everything on earth represents something in the spirit world, talked about the mother tree, the largest and tallest tree in a forest, roots connect them and support the health of all trees and plants (similar to women participating in the program) • Women received baskets of traditional foods • Continued to deepen bonds and connections with each other
Session 4. Indigenous archives at St Paul Science museum + Lunch at Owamni (traditional Indigenous restaurant)	• Women were allowed to see, touch, smell, and use artifacts in an area not open to the public • They expressed that it felt like a privilege to observe part of history: beads, pottery, animal leather works, bear, hand crafts, toys, clothing, and weapons • The experience evoked respect for ancestors, as well as sadness for lost culture; members expressed they could feel ancestral energy • Dining at Owamni is often out of reach for many Indigenous people due to the cost • Women were encouraged to dream big as they imagined their futures and reported they felt like they were “eating possibilities”
Session 5. Tobacco harvesting	• Learned how to harvest and process tobacco from red-willow, a sacred medicine used in Indigenous cultures • Discussed the importance of appreciating what nature provides throughout the seasons
Session 6. Sugar Bush (Maple sap harvest) opening ceremony	• A very powerful experience including tobacco ceremony • Opportunity to meet others from the community, share food, and hear traditional songs • Acknowledged the cognitive dissonance that can occur from participating in cultural ceremonies and then shifting back to mainstream culture
Session 7. Mahnomiin conference	• Learned about the importance of wild rice • Participated in a pipe ceremony, heard other cultures share about wild rice, learned native games and crafts • Women noted deepened connection to one another through shared learning experiences
Session 8. Medicine garden blessing	• A blessing from an Elder and traditional prayers and songs to activate the land before planting • Women felt honored to attend
Session 9. Native planting/prairie restoration	• Learned how park systems are working to return outdoor spaces to their original state and restore native plant species • Noted how access to natural resources is possible even in the metro area
Session 10. Medicine making and final celebration	• Final celebration day • Learned more about plant medicines • Received gifts acknowledging and reflecting on the year of activities

There were 10 sessions in addition to recruitment and session previews, organized around cultural practices and healthy living traditions, reconnecting the group of women to their Indigenous roots. Traditional Indigenous social norms were highlighted. For example, gift sharing, a key element in Indigenous sociality, was integrated into the sessions. Program activities were not instructional but experiential, immersing participants in an Indigenous context where they bonded with and learned from each other. The collective experience was also in honor of Indigenous ways of life [28].

Participants were recruited through Indigenous ceremonies. The Elders offered sacred tobacco to their ancestors for guidance on who to invite to the program, out of respect and gratitude. In total, RWWAK included 2 Elder-leaders, 7 consistent participants, 1 additional inconsistent attender, all of whom were 30 or older, resided in the Twin Cities metropolitan area, and came from tribes located in South Dakota, Wisconsin, or Minnesota.

### Evaluations

Evaluation for RWWAK is different from evaluations done for other existing health promotion programs. The dominant approach utilizes Western health standards as measures for program outcomes, aligning program success with improvements in scientific health indicators. This approach contrasts with the rationale of culturally relevant activities. Recognizing the incongruence between program content and its evaluation, which is prevalent among existing programs, evaluation for RWWAK aimed to achieve cultural relevance, rooted in Indigenous understanding of program effectiveness.

To explore the developmental processes of this program, a developmental evaluation framework was used, which facilitated a deep understanding of the program’s progression and supported the advisory committee in framing the program’s objectives and expected outcomes. This approach aligns with Patton’s guidelines for developmental evaluation [29].

Data were systematically gathered through direct observations and comprehensive documentation of the program’s development, guided by the insights of Indigenous Elders and community leaders. Additionally, reflective sessions were conducted with Extension program leaders and members of the advisory committee. These sessions included innovative methods, such as reflective journaling, journey mapping, and storytelling through visual arts, specifically drawings and photos, to capture rich narratives and feedback about the program’s evolution and its initial impacts.

The analysis of the collected data was performed by an internal evaluator and program stakeholders. The analysis was guided by key evaluation questions that community members considered the most important to them. All the collected data were then thematically analyzed to answer these questions. Observation notes went through open coding that resulted in a large number of themes, and then, these themes were combined into more summative ones. For visual data, such as pictures and drawings, efforts were made to identify major themes in them that would address the questions. Findings from both visual and nonvisual data were then compared and contrasted to develop a comprehensive understanding of the data as well as answers to the questions. Preliminary findings were subsequently reviewed and validated by community Elders, ensuring their alignment with community perspectives and insights. The specific evaluation questions formulated for this study and the resultant findings are detailed in Table 3.

**TABLE 3:** Evaluation results.

What program format/model emerges when a cultural community participatory approach is facilitated?
• The model emerged into a “society” of Indigenous Elder women, where sharing, relationships, and group dynamics are critical; supportive relationships are cultivated and lateral oppression is actively discouraged. • The activities encompassed a variety of culturally immersive experiences, including workshops on traditional arts and crafts, educational sessions on the use of native ingredients, hands-on opportunities in harvesting and preparing traditional dishes, shared communal eating experiences celebrating cultural cuisine, informative sessions about significant cultural ceremonies, and engaging in authentic, culture-based experiences that foster a deeper understanding of the heritage and traditions. • The model emphasized shared learning and leadership development, where members were encouraged by an Elder to share from their own wisdom and experience, and also encouraged to use and repeat the things they are learning. Through this, the advisory members developed skills to guide and mentor others.
What decisions and activities proved critical in program development?
• The program development process was fully designed and implemented by the Elders and the advisory members. Although they shared their progress with and requested administrative/logistical assistance from Extension, trust was placed in their cultural and community knowledge to design a program that honored cultural ways of learning and not influenced by dominant cultural constructs. We have organically built with *Elders* and community voices a program model that is unique and truly reflective of our Indigenous ways of teaching, learning, and living. • Interspersed overnight events enabled members to develop trusting relationships with one another, and the group dynamic benefited from these more substantial shared experiences. We joined a water protectors camp where there were people from all over the world gathered to honor and protect the water, and learn how to harvest wild rice. We stayed overnight in a yurt complete with spiders and frogs, and laughed so much that the others at the camp called us the “Cackling Aunties” and brought us gifts of maple syrup. When we went out in the canoes in the morning, despite never having harvested wild rice before, all of us felt the sense that this activity was part of our ancestral blood memory. It was spiritual and emotional. And hard physical work as well! • In addition to the planned events, time for reflection and sharing of personal struggles with support from an Elder with some training in therapeutic guidance, was essential in building the trust and intimacy among the group. The more time we have spent together, the more we have developed into a socially supportive society of *Elders.* We have cooked traditional foods together, made traditional crafts, harvested tobacco, and participated in Blessings and Ceremonies. And we have spent much time together reflecting on our roles in our families and our communities, providing each other with needed support, and developing an understanding of our roles as medicine keepers. We encourage one another to consider our ancestors, families, communities, children, and grandchildren with our lifestyles choices and decisions. We are always thinking 7 generations into the future. • Ongoing personal communications and supportive assistance between Elder-facilitators and advisory committee members was critical for supporting the advisory members’ involvement, such as providing information about resources, personally delivering program honoraria, coordinating transportation to events, and keeping them apprised of upcoming activities.
What outcomes were identified as important and significant to the members?
• Emotional outcomes: In a journey-mapping process, the advisory members shared how they quickly moved from an emotional place of nervousness and feelings of doubt and self-judgment during their recruitment and initial meeting, to a place of comfort and excited anticipation, confidence, willingness and safety in sharing vulnerabilities with one another. They reported finding purpose and success, and feeling nurtured and lucky as the program continued. As advisory members, we felt honored, and a little nervous to be involved in this program. [Elder Name] identified each of us to serve on the advisory committee by putting down tobacco, and our names came to her. We didn’t know what the year ahead was going to involve, we didn’t know each other, and we all had different connections to our Indigenous Teachings and Traditions. • Personal abilities: Some reported doubts in their personal abilities to participate in the events and activities (for example, “I don’t know what wild rice looks like”), and were surprised and pleased that they were able to successfully do so. Some reported a desire to participate in the activities with friends and family, and toward the end of the program, 2 members reported that they had invited their families to partake in one of the activities they had first experienced through the program. • Social connectedness: The socially supportive sisterhood that emerged during the program was another notable outcome from the advisory members. They reported connecting with one another socially outside of the program, and demonstrated support for one another during program events. This connection between members was even apparent at evaluation meetings with the program administrators, where members drew each other out, referenced stories in which they showed support and trust for one another, and showed kindness, compassion and respect for one another. We immediately bonded as sisters, and we felt so much support. We learned to render bear grease and we made traditional teas. When the day was over none of us wanted to leave; we sensed a deep connection to one another through learning Traditional Healing ways together, and felt honored that this medicine making was part of our traditional culture and way of knowing as *Elders.* • Health: Advisory members acknowledged the positive influence of the physical activity and healthy foods offered through the program. They also made note of the fact that they were pleased to have had positive experiences without the influence of alcohol. They pointed out the value of having enough time together to shed the stresses of their day and spend time reflecting and sharing to unload and heal from their stress, trauma, and life realities. • Connection to culture: Several advisory members described participation in the program as life-changing due to its ability to help them internalize their Elder-identity as medicine keepers within their culture and ancestry. I was asked to sum up our experience and what it meant to me. In a word: Restorative. When you are reminded of who, what, and where you come from, you begin to better understand who you are and your place in the many circles you occupy. Including our first languages, practicing prayer, and making offerings to the Creator, ancestors, and Mother Earth before we take is protocol. Putting our Indigenous words to the activities and plant medicines made it alive and meaningful.So this is what we already knew. But profoundly, it may have been a very long time or even the first time we were invited to do so. That invitation allowed us the space to give our time and attention and receive the incredible support needed as we went down this path together. We are restoring the health and hope that comes from living life in balance, as it was intended for us. In Ojibwemowin, we say Bimaadiziwin, The Good Life, or going in a good way. That is the direction we are pointed in.
What aspects of the program development process are most important to replicate when establishing a program moving forward?
• Facilitator: The group is preferably coled by Indigenous Elder women with deep knowledge of community, resources, healing, social/emotional guidance, and cultural ways as well as ability to coordinate and manage logistics. Facilitators must have the ability to serve as guides and mentors and to support members in reconnection with their cultural heritage and growth in their roles and responsibilities as medicine keepers. • Recruitment: For this development project, one of the Elder-facilitators recruited the advisory team. This was done through laying down tobacco to receive guidance from the Creator in the selection of each participant, and then formally inviting them to serve as advisory members. The invitation from an Elder was an important motivator for participation by the advisory team. Even if future members are referred by community sources, an invitation to become a member will be issued by an Elder-facilitator to support and motivate their participation. • Orientation: Introduction to this program was accomplished through sharing a meal and receiving gifts. The first and primary message to the advisory members was that this society is about reconnection to culture, finding identity and assuming roles and responsibilities as Elders and medicine women. Additionally, it is about learning, growing and uplifting one another within a society of Indigenous women and intentionally shedding tendencies toward lateral oppression. It is a place to share pain and vulnerabilities, to let go of fear, and be able to walk in 2 worlds and move forward through life’s discomfort with love and grace. This orientation time together laid an important foundation for ongoing interactions and activities. Note: advisory members were not told that this was a diabetes prevention program, nor will this descriptor be a priority for future members. • Activities: For this project activities have taken place at least bimonthly. All activities have been experiential, culturally-based activities with time for sharing and reflection. This frequency has seemed appropriate to maintain connection as well as make participation manageable for women with busy work and personal schedules. • Essential elements: ○ Gifts/compensation: Society members are honored for their presence through gifts and compensation. Gift-giving is a cultural tradition showing respect and appreciation. The gifts given to advisory members have been cultural items (tobacco, sage) Indigenous foods (maple and berry syrups, wild rice) and items that have enabled members to follow healthy cultural practices (bento boxes to bring healthy foods and meals to ceremonies and pow-wows). Compensation is necessary for the time commitment required for participation, during which they may not be able to work, they must arrange for care for household members, etc. ○ Healthy cultural foods/meals: Food/meals have been an essential element in program activities. Culturally, food is a joiner, a leveler, an expression of honor and gratitude. Food must be in abundance, must be culturally congruent, and must be offered in the spirit of unobligated sharing and a gift. Food is reflective of family time, releasing the weight of the day, nourishing body, mind, and soul. It calms, brings a sense of needs being met, and shows caring. Sharing of food is always accompanied by ceremonies of gratitude and recognition of ancestral heritage. ○ Physical activity*:* Physical activity plays an integral role in planned activities, such as ricing, tobacco harvesting, attending outdoor ceremonies, and walking through museum archives, but is not explicitly called out as such. ○ Place-based*:* members and facilitators live in the same geographic area to facilitate connection and common context. Having members within a geographic region has enabled them to connect with each other for support such as ride-sharing, and has allowed Elder-facilitators to have in-person connections and communications between activities with the members. ○ Time*:* Activities are a minimum of a ½ day duration, with some activities involving overnight stays. This duration has been praised and appreciated by advisory members. It creates time for connection, trust-building, reaction, and reflection. It allowed for letting go of prior activity, sharing of joys, pain, and vulnerabilities and receiving support from Elders and other society members. ○ Cultural traditions/Ceremonies*:* Cultural traditions and ceremonies (e.g., offering tobacco, spirit plates of food, etc.) are practiced throughout activities, and Elder-facilitators mentor and share cultural knowledge and wisdom. • Dosage: Consistent with the Indigenous cultural construct of the circular nature of time, the medicine wheel of holistic health, and the universe, membership and participation in this society can begin at any juncture and does not have a time limit. Emerging Elders may participate as long as they are available and it is useful for them, and they may ultimately become leaders for society activities as they assume their identity, roles, and responsibilities as Elders.

## Results

Results of the evaluation are centered around four evaluation questions consistent with the theory of change of RWWAK. These questions address the overall format of RWWAK, key decisions, activities, and outcomes. The results depict a full picture of RWWAK, examining its process and outcomes, and provide a glimpse into future iterations. Although details surrounding each of the dimensions are discussed in Table 3, a summary below illustrates key messages the evaluation has delivered.

One of RWWAK’s key achievements was community building, and emphasis was placed on the socioemotional well-being of its participants. The culturally safe and supportive environment helped them to develop positive relationships with each other as they explored and internalized their Indigenous role as medicine keepers. RWWAK fundamentally motivated its participants, helping them reconnect with their cultural roots to foster healthy lives for themselves and others.

Additionally, the evaluation results point to key factors and arrangements that ensured the Indigenous authenticity of the project. Facilitation by respected Indigenous Elders, gathering at meals of traditional foods, and engaging in Indigenous ceremonies and customs such as smudging and gift-giving all provided an authentically Indigenous context for the project, leading to the outcomes discussed above and in Table 3. These factors and arrangements are not universal or normative as RWWAK was conducted for a small number of participants and insights obtained from it may not apply in other Indigenous contexts. The results are evidence that approaches to diabetes prevention exhibited through RWWAK deserve more attention.

## Discussion

The effectiveness of this Two-Eyed Seeing approach has been evident through enhanced opportunities for learning throughout the program development, increased knowledge about healthy living and diabetes prevention, and elevated respect for and pride of cultural knowledge and approaches. This community-driven program model differs from mainstream health program development and implementation and emphasizes the following:

1) Focusing on health and wellness in general, rather than diabetes prevention may resonate more with members.
2) Connecting members to their cultural roots can strengthen active participation in healthy behaviors.
3) Embracing culture generates health and supportive relationships generate health.
4) Sharing and modeling knowledge and wisdom is preferred over an expert-model educational approach; there is an acknowledgment that all bring expertise.
5) Focusing on behavioral change and BMI change is not meaningful.
6) Providing safe and open relationships at the early stages of the program, free from influences of dominant culture, can allow for deeper engagement.
7) Building trusting relationships between program leads, community leaders, and community members is the foundation of developing and sustaining the program.

Evaluation results of RWWAK demonstrate that participants were guided to reconnect with their cultural roles of medicine keepers, and the bonds they established with each other provided significant socioemotional support, encouraging them to prioritize their own health, the health of their own communities, and mentor younger generations. All of these developments support the theory of change underpinning RWWAK. The close alignment of theory and practice resonates with the growing literature demonstrating that culture and community connections are important yet overlooked components for successful health promotion programs for Indigenous communities [12].

Additionally, RWWAK contributes to understanding why culture and community connections matter. It helped Indigenous community members see beyond their colonized realities and reconnect with their authentic but lost way of life. Such reconnections are healing as they start to reconceptualize “a healthy life,” moving away from Western scientific standards and toward a more holistic notion of health involving multiple dimensions and generations. The program required investment in trust-building and courage from all involved to question and address barriers related to institutional practices and systems, staff credentials and roles, and funder expectations (for example, controls on expenditures, content and curriculum development expertise, and planning templates and timelines).

An insight RWWAK generated was that to deeply understand such reconnections and healing requires researchers to go beyond Western scientific paradigms as these paradigms define health in a way that is uncommunicative to Indigenous paradigms. Healing taking place in the Indigenous worldview can only be understood through Indigenous eyes. To support such an Indigenous worldview does not suggest refusal of Western scientific paradigms. What is needed is the utilization of the Two-Eyed Seeing approach.

Overall, RWWAK demonstrates the potential of disrupting the dominant culture systems. Although mainstream programs have demonstrated success in physical health improvements, they often overlook the cultural dimensions critical to sustained health behavior changes. Traditional ecological approaches offer valuable insights into creating more effective and inclusive chronic disease prevention strategies. Incorporating more culturally aligned perspectives in health promotion programs can help address the limitations of current mainstream approaches and enhance long-term effectiveness and accessibility. Shifting from a mainstream program development model to one that centered community and culture required trust and courage. Institutional and funder roles and systems have been upended through this approach. Working in this way will continue to result in new relationships, new ways of working, new ways of reaching out to the community, and increased engagement with holistic health programs from and for the community. Given the small number of participants in RWWAK, insights from the project may not have applicability across Indigenous contexts, and this limitation serves as an invitation to further exploring approaches to health promotion for Indigenous communities that are authentic, organic, effective, and culturally sustaining.

## Author contributions

The authors’ responsibilities were as follows – BP, KW, TA, HK, and EJMB: designed research (project conception, development of overall research plan, and study oversight); KW, TA, HK, BP, and EJMB: conducted research (hands-on conduct of the experiments and data collection); KW, TA, HK, BP, EJMB: analyzed data or performed statistical analysis; BP, KW, TA, and HK: wrote paper (only authors who made a major contribution); BP, KW, TA, HK, and EJMB: had primary responsibility for final content; and all authors: have read and approved the final manuscript.

## Data availability

Data described in the manuscript will not be made available because it contains Indigenous references that are considered culturally sacred and private.

## Funding

Program development was funded by the Centers for Disease Control and Prevention WiseWoman Program via Minnesota Department of Health SagePlus, and by the Supplemental Nutrition Assistance Program–Education via Minnesota Department of Human Services. The supporting sources had no involvement or restrictions regarding publication.

## Conflict of interest

TA reports financial support and travel provided by Centers for Disease Control and Prevention (via MN Department of Health) and USDA-Food and Nutrition Service (via MN Department of Human Services). KW reports financial support provided by Centers for Disease Control and Prevention (via MN Department of Health) and USDA-Food and Nutrition Service (via MN Department of Human Services). HK reports financial support provided by Centers for Disease Control and Prevention (via MN Department of Health) and USDA-Food and Nutrition Service (via MN Department of Human Services). EJMB reports financial support and travel provided by Centers for Disease Control and Prevention (via MN Department of Health) and USDA-Food and Nutrition Service (via MN Department of Human Services). BP reports financial support was provided by USDA-Food and Nutrition Service (via MN Department of Human Services).
